# A treatment-refractory aggressive MDS-MLD with multiple highly complex chromosome 5 intrachromosomal rearrangements: a case report

**DOI:** 10.1186/s13039-022-00630-0

**Published:** 2022-12-06

**Authors:** Ramakrishnan Sasi, Jamie Senft, Michelle Spruill, Subit Barua, Sam Dougaparsad, Jeffrey A. Vos, Peter L. Perrotta

**Affiliations:** 1 Department of Pathology, Anatomy and Laboratory Medicine, West Virginia University, Morgantown, WV 26506 USA; 2 Biodiscovery, El Segundo, CA 90278 USA; 3 Essentia Health – St. Mary’s Medical Center, Duluth, MN 55805 USA

## Abstract

**Background:**

A patient with a myelodysplastic neoplasm exhibited a karyotype with multiple complex chromosome 5 rearrangements. This patient appeared to have a catastrophic cytogenetic event that manifested as a treatment-refractory aggressive form of disease, which lead to patient demise within one year. Both the clinical presentation and disease course were unusual based on the medical history and morphologic findings. Such cases of myelodysplastic syndrome with multilineage dysplasia (MDS-MLD) with complex abnormalities are not reported in the literature.

**Case presentation:**

The patient was a 62-year-old female who presented with pancytopenia and dyspnea. The morphologic appearance of the peripheral blood smear and bone marrow biopsy, along with flow cytometric findings, favored the diagnosis of MDS-MLD unclassifiable. Myelodysplastic syndrome (MDS) with multilineage dysplasia (MDS-MLD), is an MDS characterized by one or more cytopenias and dysplastic changes in two or more of the myeloid lineages (i.e., erythroid, granulocytic, and megakaryocytic). The bone marrow, in particular, showed prominent dysplasia, including the presence of atypical megakaryocytes with small hypolobated morphology reminiscent of those typically seen in MDS with isolated 5q deletion. Cytogenetic analysis, including interphase and metaphase FISH, karyotype and SNP chromosomal microarray were performed, as well as DNA sequencing studies. Cytogenetic analysis showed a very complex karyotype featuring multiple 5q intrachromosomal rearrangements including a pericentric inversion with multiple interspersed deletions and monosomy 7. FISH studies showed a partial deletion of the *PDGFRβ* gene, and SNP chromosomal microarray and targeted panel-based sequencing identified biallelic loss of function of the *TP53* gene. Based on the pathologic findings, the patient was treated for MDS but did not respond to either lenalidomide or azacitidine.

**Conclusion:**

The genetic changes described, in particular, the complex intrachromosomal rearrangements of chromosome 5, suggest the occurrence of a sudden catastrophic event that led to an aggressive course in the patient’s disease. Conventional karyotyping, metaphase and interphase FISH, SNP chromosomal microarray and NGS helped to identify the complex genetic changes seen in this case. This highlights the importance of utilizing a multimodality approach to fully characterize complex chromosomal events that may significantly impact disease progression, treatment and survival.

## Background

Myelodysplastic neoplasms (MDS), formerly known as myelodysplastic syndromes comprise a group of clonal hematopoietic stem cell malignancies involving one or more myeloid lineages that result in abnormal cellular maturation [1, 2]. Clonal chromosomal abnormalities are observed in approximately 50% of de novo and up to 90% of therapy related MDS patients [3–5]. Monoallelic interstitial or terminal deletions of the long arm of chromosome 5 [del(5q)] is a recurrent cytogenetic abnormality common to a category of MDS sometimes referred to as 5q- syndrome [4, 5]. This category of MDS has a relatively favorable prognosis with low risk of progression to AML as compared to other types of MDS [2, 3]. Previous cytogenetic and molecular studies have reported two small commonly deleted regions of chromosome 5q that can cause the loss of a contiguous region resulting in haploinsufficiency of more than 40 candidate genes including *RPS14*, which encodes a component of the 40S ribosomal subunit that is associated with normal blood cell development [5–7]. The mechanism causing erythroid failure appears to involve decreased expression of the ribosomal protein S14 (*RPS14*) gene and upregulation of the *TP53* pathway through ribosomal stress [6, 7]. Studies have shown that partial loss of function of the ribosomal subunit protein *RPS14* mimics the disease in normal hematopoietic progenitor cells [8, 9]. Recent molecular studies have shown that allelic haploinsufficiency for several genes located on 5q, including RPS14 and *EGR1*, are responsible for both the hematologic phenotype and the sensitivity to lenalidomide in this MDS subtype [4, 5, 8].

Clinical data and literature suggest most patients with 5q- syndrome (as the sole change) have a good prognosis [2]. However, a subset of MDS patients with 5q deletion may undergo clonal evolution resulting in the formation of complex karyotypes [10]. A complex karyotype, defined as at least three independent clonal aberrations, is detected in approximately 10–15% of MDS patients and is associated with a short median survival of less than 12 months and a high risk of AML transformation [10–12]. Loss of function mutations of the *TP53* gene and monosomy7/7q deletions are common secondary changes associated with clonal evolution [5, 11, 13].

Among the multiple clonal abnormalities identified in the bone marrow, cytogenetic studies revealed a partial deletion of *PDGFRβ*. The PDGFRβ rearrangements seen in MDS/MPN are rare but delineate a distinct type of myeloid neoplasm with characteristic clinicopathologic features. Several rare gene fusions involving *PDGFRβ* have been described in patients with chronic myeloproliferative disorders (MPD), myelodysplastic/myeloproliferative syndromes (MDS/MPD) and AML [1]. These are often associated with eosinophilia and splenomegaly and may respond to imatinib mesylate therapy [1]. In this study, FISH study showed deletion of the 3′ and most of the coding regions of PDGFR*β gene* leading to loss of its kinase activity. Therefore, these results predict the lack of response to usual TK1 inhibitor therapies including Gleevec.

In this report, we describe a patient with highly complex intrachromosomal rearrangements of chromosome 5 including multiple deletions and a large pericentric inversion present in three karyotypic clonal populations. We used conventional karyotype analysis, metaphase and interphase FISH studies, SNP chromosomal microarray analysis and next-generation sequencing (NGS) to characterize the genetic changes seen in the 5q region and to better understand the patient’s pathological findings and disease course.

## Case presentation

A 62-year-old female presented with pancytopenia and dyspnea. The patient’s history revealed anemia and treatment for iron deficiency. The peripheral blood smear appearance, complete blood count (CBC) parameters and flow cytometric findings were concerning for MDS. The bone marrow biopsy showed a hypercellular marrow (60–70% cellularity) with mild dysplastic changes most prominently noted in the megakaryocytes, moderate myelofibrosis, and less than 1% blasts. Erythroid precursors were relatively increased, and myeloid maturation appeared left shifted. The initial findings favored the diagnosis of MDS/MLD, unclassifiable. However, the morphologic features of the bone marrow core biopsy, in particular, the presence of atypical megakaryocytes with small hypolobated morphology were reminiscent of those typically seen in MDS with isolated 5q deletion. Based on the pathologic findings, the patient was treated for MDS but did not respond to lenalidomide or azacitidine. Her disease progressed and she died within one year of initial presentation.

## Results

### Cytogenetic analysis

Bone marrow aspirates from the patient were cultured unstimulated overnight and for 48 h according to routine cytogenetic protocols. Chromosome analysis was performed on twenty G-banded metaphase cells and the resulting karyotypes were described according to the International System for Human Cytogenetic Nomenclature (ISCN 2020) [14].

Initial bone marrow karyotype analysis showed a complex karyotype with three cells lines 46,XX,der(5)del(q15q32)inv(p13q32)[3]/46,idem,add(10)(p13), − 14, − 15, − 20, + 3mar[10]/45,idem, − 7, − 18, + mar[3]/46,XX[4].

**Fig. 1:**
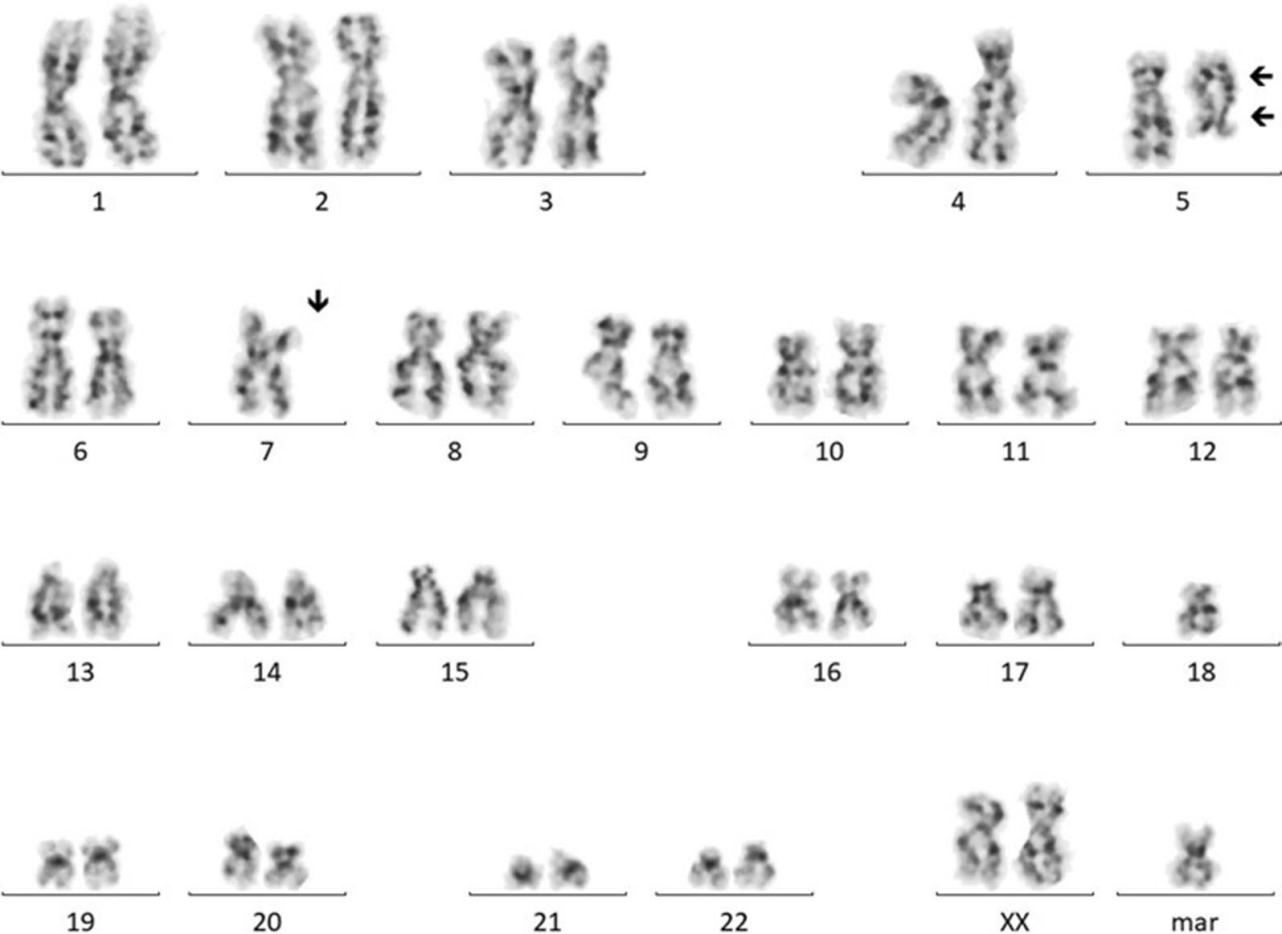
Karyotype example of clone #3. Clonal aberrations include the der(5)del(5)(q15q32)inv(5)(p13q32), monosomy 7 and a marker chromosome

The stemline featured multiple 5q rearrangements including a large deletion and an inversion, with sidelines having multiple additional structural and numerical abnormalities including monosomy 7 (Fig. 1).

### Fluorescence in situ hybridization (FISH) analysis

Interphase and metaphase FISH studies were performed on bone marrow aspirate preparations by standard methods. Briefly, interphases and metaphases were prepared from the bone marrow sample and hybridized with fluorescently labelled DNA probes purchased from commercial vendors (Abbott Molecular and Cytocell).

**Fig. 2:**
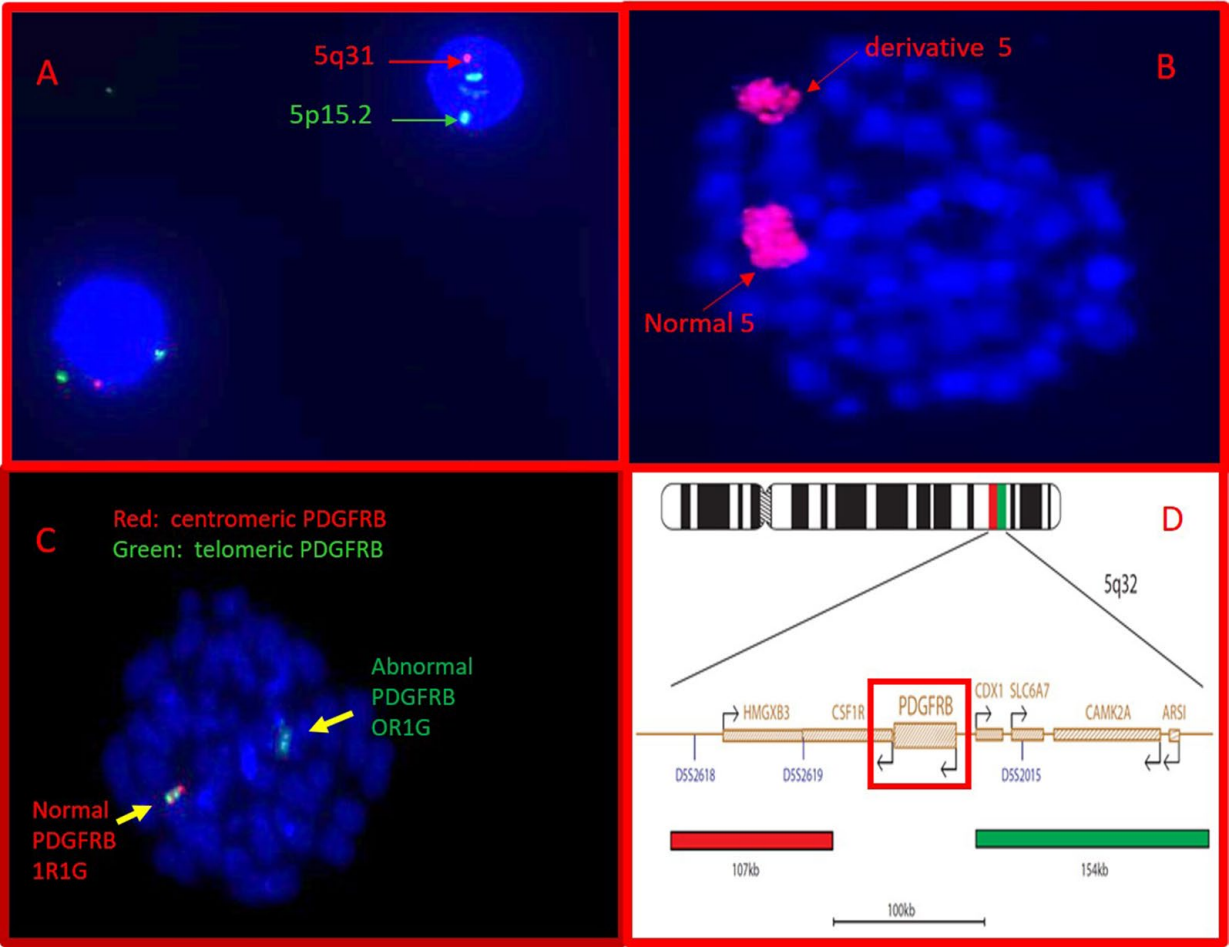
Bone marrow interphase and metaphase FISH. **A** 5q deletion probe (Abbott Molecular): a single red signal indicating a 5q31 (EGR1) deletion and two normal control 5p15.2 (D5S23/D5S721, green) signals. **B** Whole-chromosome 5 paint probe (Abbott Molecular, red) on bone marrow metaphase preparations showing intense hybridization to both the normal and derivative 5, with no hybridization to any other chromosome. **C** Hybridization with 5q32 PDGFRB break apart probe (Cytocell) showing deletion of the centromeric side of the PDGFRB probe (red), while the telomeric side (green) appears intact. **D** PDGFRB probe map (adapted from ogt.com)

**Fig. 3:**
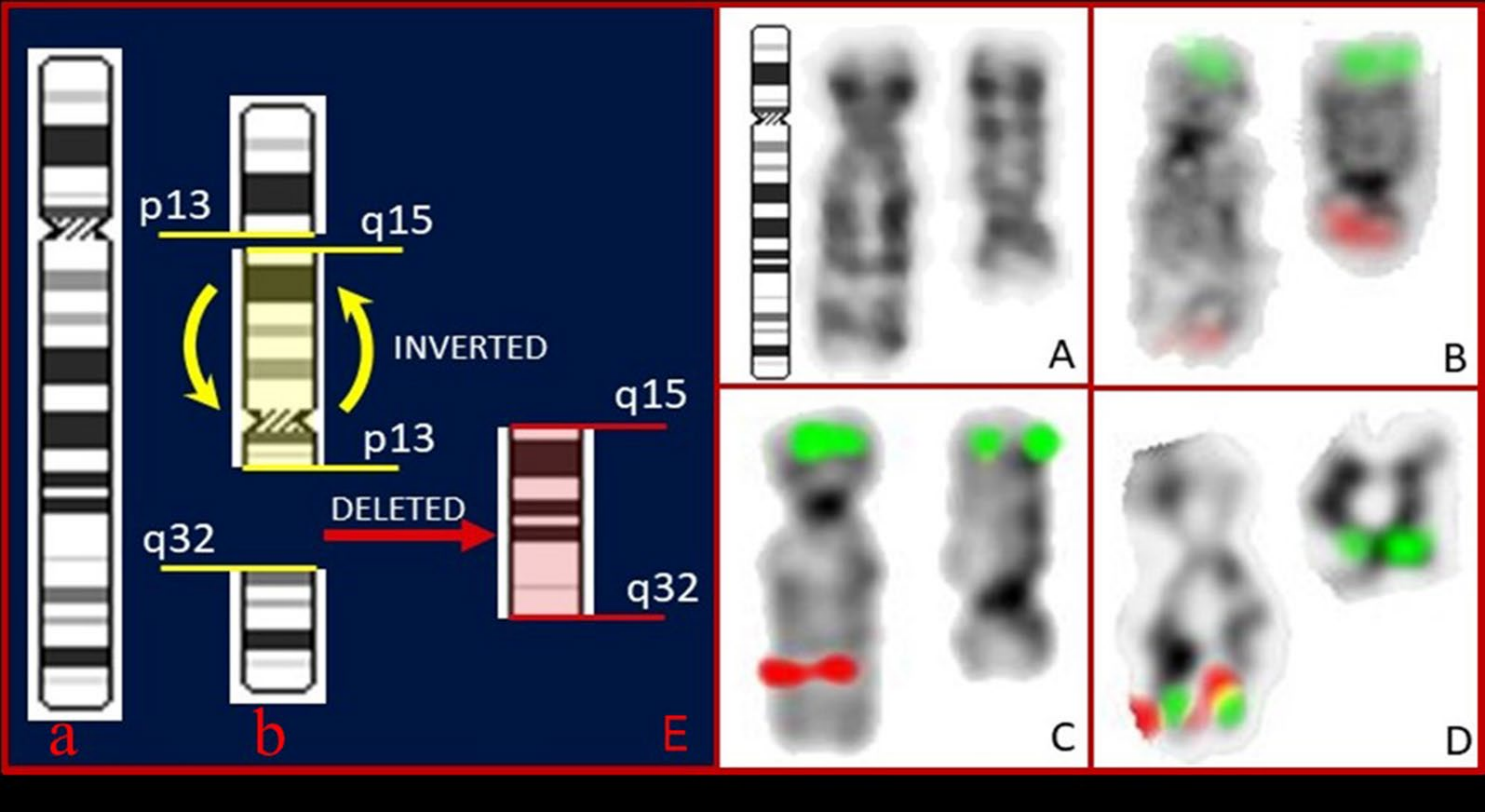
5q inversion and deletion. **A** G-banded normal chromosome 5 and der(5)del(q15q32)inv(p13q32). **B** Metaphase FISH using telomere (5p—green/5q—red) probes (Abbott Molecular) on inverted DAPI-banded chromosomes (normal and abnormal). **C** Metaphase FISH using 5q31 (red)/D5S23/D5S721 (green) probe (Abbott Molecular) on inverted DAPI-banded chromosomes (normal and abnormal). **D** Metaphase FISH using PDGFRβ break apart probe (Cytocell); 5′(green)/3′(red). Loss of 3′ centromeric probe (red) shown on inverted DAPI-banded abnormal derivative chromosome 5. **E** Ideogram of normal 5 (a) and der(5) (b) showing excision of a large segment of DNA located on 5q15q32 (red), followed by a large pericentric inversion inv(5)(p13q32) (yellow)

Summaries of interphase and metaphase FISH studies are shown in Figs. 2 and 3. FISH studies showed the deletion of 5q31 (*EGR1*) (Figs. 2A, 3C). Metaphase FISH using the *PDGFRβ* break apart probe (Figs. 2C, D, 3D) shows the partial deletion (centromeric side) of the *PDGFRβ* gene as revealed by the loss of the proximal (red) signal shown on the inverted DAPI-banded abnormal derivative chromosome 5. This result suggests that most coding regions of the PDGFR*β* gene are lost and only a small region of the 5′ end of the probe remains. Therefore, it appears the kinase activity of the partially deleted *PDGFRβ* gene would be lost rendering this patient’s disease refractory to Gleevec or related new generation TK1 inhibitor-based treatments.

The chromosome 5 paint study clearly demonstrated the derivative chromosome 5 was comprised exclusively of chromosome 5 material (Fig. 2B). Figure 3 summarizes the FISH findings using metaphase FISH and ideograms to illustrate the complex rearrangements involving chromosome 5. The ideograms show a normal chromosome 5 (Fig. 3a) and the der(5) (Fig. 3b) with the large segment of DNA located on 5q15q32 excised in red and a large pericentric inversion inv(5)(p13q32) shown in yellow. The inverted DAPI-banded chromosomes with the individual FISH probes that were used to help elucidate the der(5) abnormalities are seen in Fig. 3B–D.

### SNP microarray copy number analysis

Whole genome SNP microarray analysis was performed on genomic DNA extracted from the bone marrow aspirate using a Qiagen kit/QI Cube System. The SNP chromosomal microarray array was performed using the CytoScan^®^ HD (Affymetrix, Santa Clara, CA) system according to the manufacturer’s instructions. This array contains approximately 2.695 million copy number markers including single nucleotide polymorphisms (SNP)-based oligonucleotides and non-polymorphic oligonucleotides.

**Fig. 4:**
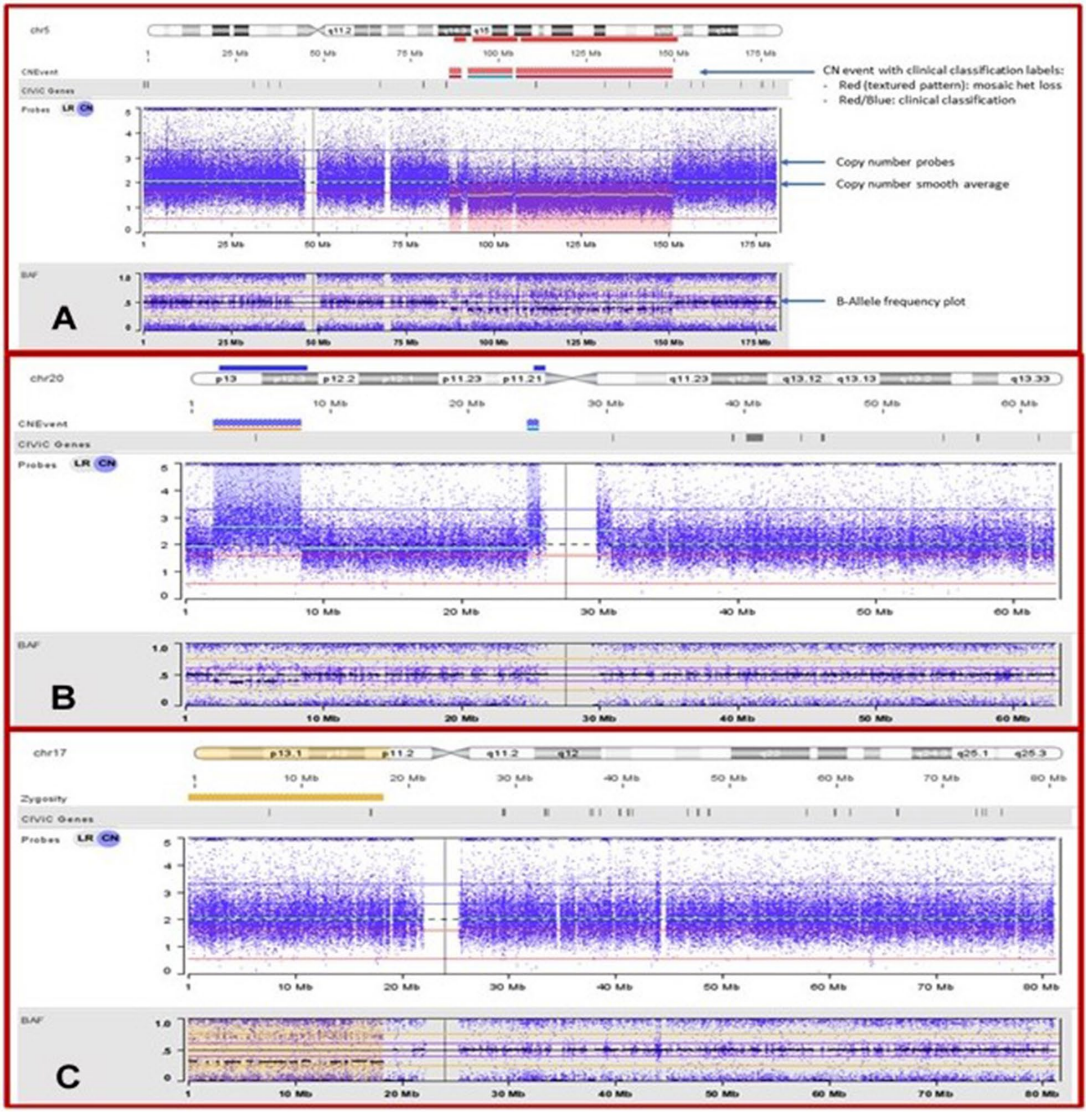
CNV, AOH detection by SNP Microarray. CEL data files generated by the Affymetrix scanner were directly analyzed using NxClinical 6.1 (BioDiscovery, LLC) for copy number variation (CNV) and copy neutral loss of heterozygosity (CN-LOH) variants at an analytical resolution of 25 kb and 3 Mb, respectively. Clinical classification codes: Red = pathogenic, Orange = likely pathogenic, Blue = VUS. **A** Chromosome 5 showing the 3 mosaic deletions. **B** Chromosome 17 showing 17p13p11.2 LOH. **C** Chromosome 20 showing the likely pathogenic duplication

**Fig. 5:**
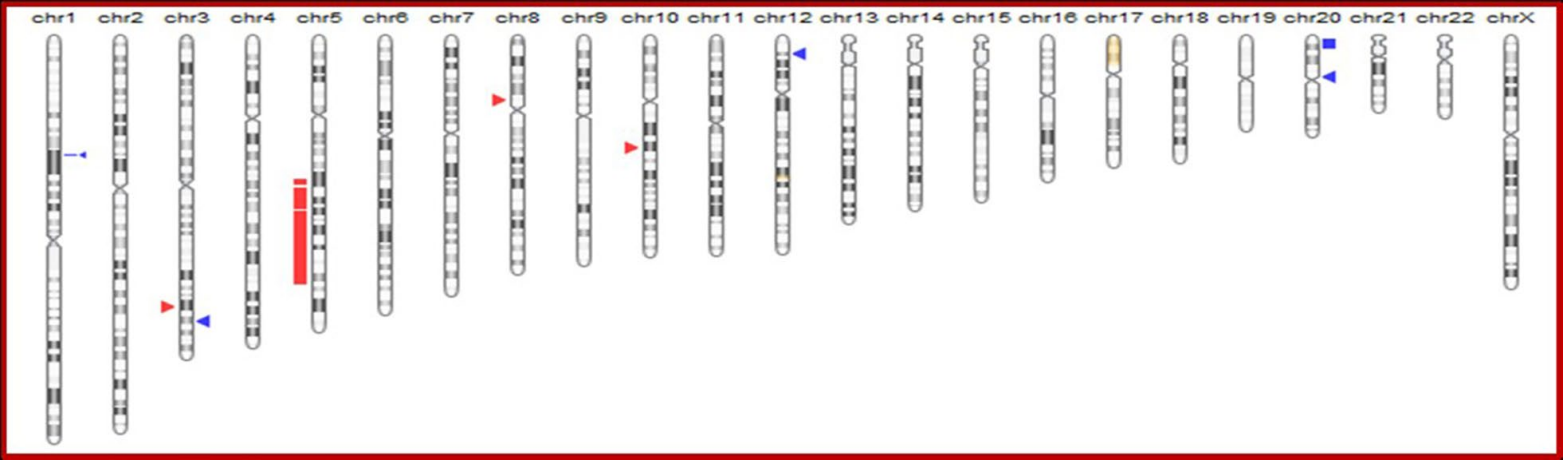
Ideogrammatic representation of all copy number (CN) gains (blue), losses (red) and copy neutral LOH (yellow) detected by the microarray

The SNP microarray data were analyzed using NxClinical software (Figs. 4, 5, Table 1. BioDiscovery, El Segundo, CA), revealing at least three separate deletions interspersed in the 5q14q32 region without any duplications. The total deleted region comprised 61.16 Mb (Table 1). All deletions were found in the same copy number state suggesting they originated as part of a single catastrophic event. The 5q14.3 and 5q21.3q33.1 were identified as pathogenic variations (Fig. 4A). The only other copy number change observed by SNP microarray was an 18.9 Mb duplication in the 20p13p12.3 region; this duplication was considered likely pathogenic (Fig. 4B). An ideogrammatic representation of all copy number (CN) gains (blue), losses (red) and copy neutral LOH (yellow) detected by the SNP microarray study results are summarized (Fig. 5, Table 1). Other changes identified by karyotype analysis were minor clones and fell below the level of detection of the microarray test.

**Table 1:** Data analysis: CNV, AOH detection by SNP microarray

Event	Aberrant cell fraction %	Estimated copy number	Estimated copy number of tumor fraction	ISCN nomenclature	Length Mb	Gene count	CIVIC gene count	COSMIC census tier I count
CN loss	44.3	1.49	1	5q15q21.3(92645401_102210820) × 1 ~ 2	12.87	52	0	0
CN loss	43.9	1.49	1	5q14.3(87333557_90841423) × 1 ~ 2	3.51	19	0	0
CN loss	43.4	1.51	1	5q21.3q33.1(106408771_151202965) × 1 ~ 2	44.79	452	5	5
CN gain	54.4	2.65	3	20p13p12.3(2014047_8404702) × 2 ~ 3	6.39	88	1	0
AOH	38.5	2/AOH	2/AOH	17p13.3p11.2(1_18289923)x2 mos hmz	18.29	394	9	9

CEL data files generated by the Affymetrix scanner were directly analyzed using NxClinical 6.1 (BioDiscovery, LLC) for copy number variation (CNV) and copy neutral loss of heterozygosity (CN-LOH) variants at an analytical resolution of 25 kb and 3 Mb, respectively

In addition, SNP microarray analysis showed a 17p mosaic copy neutral LOH that included the 17p region where the *TP53* gene resides (Fig. 4C). MDS/AML targeted panel exon sequence analysis performed at a reference laboratory reported a single point mutation (c.707A > G (p.Y236C) in the *TP53* gene (data not shown). *TP5*3 (p.Y236C) lies within the DNA-binding domain of the *TP*53 protein [15]. This missense variant occurred in the DNA-binding domain at a mutational hotspot reported in various tumors as a somatic mutation or as a germline variant in Li-Fraumeni syndrome. In alignment with the SNP microarray finding of mosaic copy neutral LOH at chromosome 17p, the sequencing data confirmed somatic biallelic loss of function (LOF) of the *TP53* gene in this patient.

## Discussion

Results of the bone marrow karyotype and FISH studies demonstrated highly complex intrachromosomal rearrangements of chromosome 5. The chromosomal stemline had a der(5)del(q15q32)inv(p13q32) as the sole abnormality. FISH studies using telomere-specific probes confirmed the presence of the pericentric inversion (Fig. 3B).

Both MDS/MPN metaphase and interphase FISH panel studies showed loss of most of the coding regions leaving only a remnant of the 5′ end of the PDGFRβ gene. This explains the lack of clinical and morphologic changes typically seen in PDGFRβ-related myeloproliferative disease. Based on WHO classification guidelines, we did not designate this case as an MPN associated with PDGFRβ rearrangement due to the lack of a fusion gene and we did not anticipate a favorable response to imatinib-related therapy.

Deletions of 5q are the only genetic abnormalities that truly define a specific MDS subtype [16, 17]. Most patients with isolated del(5q) MDS can remain in a chronic stable condition for many years [2, 3], with a majority of patients dying from complications of MDS without transforming to AML [4, 5]. Therefore, some patients require tailored MDS-specific treatment(s).

In a previous report of a Ph-negative CML-like patient, one year following diagnosis and treatment, the disease progressed to a myeloid blast crisis. Multicolor banding studies revealed a complex interchromosomal translocation between two chromosome 5 homologues as the sole abnormality resulting in del(5)(q21;q23) [18]. This report, much like the case we are presenting, shows the importance of using a multimodality approach for detailed characterizations of complex cytogenetic aberrations.

An unusual case of MDS with a paracentric inv(5)(q15q33) as the only observed abnormality was recently reported in a patient undergoing routine quarterly monitoring; this patient did not require treatment [19]. Another patient was previously described having complex intrachromosomal rearrangements of chromosome 5, including a paracentric inversion [20]. In this case, the chromosome 5 rearrangements progressively evolved into three sequential clones. This patient had an initial good response to lenalidomide, but treatment was stopped after a year due to adverse effects with subsequent disease progression and the disease advanced to RAEB-2 within 6 months [20].

There are two main routes of cytogenetic clonal evolution reported in MDS with 5q deletion [10]. The majority of MDS patients undergo stepwise accumulation of cytogenetic events over a long period of time. However, a few patients, like the one we present in this case report, may have undergone an one-time catastrophic event reminiscent of chromothripsis [21]. Chromothripsis is a sudden catastrophic event in which one or more chromosomes are shattered or pulverized, and subsequently stitched back together in random order to form one or more derivative chromosome(s) with complex rearrangements and loss of heterozygosity [21, 22]. The numerous chromosomal variants characteristic of both complex karyotypes and chromothripsis portend a very poor prognosis as seen in our patient. Since chromothripsis produces highly complex genomic aberrations, its reliable detection requires a comprehensive approach that combines molecular analysis, FISH, and classical cytogenetic methods.

Whole genome SNP microarray analysis revealed at least three separate deletions interspersed in the 5q14q32 region without any duplications. These deletions encompassed approximately 315 OMIM^®^ genes including the critical *EGR1* and *RPS14* genes. All of the 5q deletions were found in the same copy number state, suggesting they originated as part of a single event (Table 1). The only other aberration seen on microarray analysis was a 20p13p12.3 (6.4 MB) duplication. The 5q FISH and microarray results indicated a mosaic state in the bone marrow sample comprised of approximately 40% neoplasm. Mosaic deletions of 5q (45–50%) including *EGR1* and *RPS14* are generally sensitive to lenalidomide according to NxClinical software analysis.

In addition to the 5q deletions, SNP microarray analysis showed 17p mosaic copy neutral LOH. This, along with the finding of a pathogenic missense variant in the *TP53* gene (hg19: chr17:7577574T > C); NM000546.5(TP53): c.707A > G, p.(Tyr236Cys), suggested a unique mechanism led to the formation of a biallelic LOF for the *TP53* gene. Loss of heterozygosity of the 17p region harboring *TP53* is a common genetic event in cancer and is known to be involved in the somatic loss of wild-type alleles in many inherited cancer syndromes [23]. The wider involvement of LOH is a feature in both heritable and sporadic cancers and is often considered as evidence for the existence of tumor suppressor gene(s) in the region of LOH.

This study reaffirms a multimodality approach is required to understand some of the genetic changes encountered in MDS, like those seen in this case. It is presumed that residual wild-type *TP53* activity in patients with mono-allelic deletion/LOF is sufficient to maintain chromosome stability; however, bi-allelic hits to *TP53* have been shown to be strongly associated with high-risk features such as complex karyotypes as seen in this patient [10].

## Conclusions

We encountered a patient with multiple deletions localized to the 5q region, along with a pericentric inversion and disruption of the *PDGFRβ* gene, abnormalities not previously reported in the literature. The highly complex nature of the derivative chromosome 5, along with the pathogenic missense *TP53* variant, 17p copy neutral LOH, and monosomy 7 (minor clone likely below the microarray LOD), were associated with a treatment-resistant and aggressive clinical course. The genetic changes suggested chromothripsis in which a sudden catastrophic event led to multiple chromosomal rearrangements in chromosome 5. This case report therefore highlights the need for employing a multimodality approach, to include conventional karyotyping, metaphase and interphase FISH studies, SNP chromosomal microarray analysis and NGS, when analyzing specimens with highly complex chromosomal abnormalities. The dramatic changes observed in the behavior of this patient’s disease furthermore illustrate the significance and necessity of fully characterizing clonal abnormalities which impact disease progression and may offer insight into clinical management.

## Data Availability

Data from this case report is unfortunately not available, due to privacy considerations. Data sharing is not applicable to this article, as no datasets were generated or analyzed during the current study.
